# The role of alcohol control policies in the reversal of alcohol consumption levels and resulting attributable harms in China

**DOI:** 10.1016/j.alcohol.2024.07.002

**Published:** 2024-12

**Authors:** Jürgen Rehm, Kevin Shield, Ahmed S. Hassan, Ari Franklin

**Affiliations:** a Institute for Mental Health Policy Research, Centre for Addiction and Mental Health, 33 Ursula Franklin Street, Toronto, Ontario, M5S 2S1, Canada; b Campbell Family Mental Health Research Institute, Centre for Addiction and Mental Health, 33 Ursula Franklin Street, Toronto, Ontario, M5S 2S1, Canada; c PAHO/WHO Collaborating Centre at CAMH, Toronto, Canada & WHO European Region Collaborating Centre at the Public Health Institute of Catalonia, Roc Boronat Street 81 - 95, 08005, Barcelona, Catalonia, Spain; d Dalla Lana School of Public Health, University of Toronto, 155 College Street, 6th Floor, Toronto, Ontario, M5T 3M7, Canada; e Department of Psychiatry, Faculty of Medicine, University of Toronto, 250 College Street, 8th floor, Toronto, Ontario, M5T 1R8, Canada; f Faculty of Medicine, Institute of Medical Science, University of Toronto, Medical Sciences Building, 1 King's College Circle, Room 2374, Toronto, Ontario, M5S 1A8, Canada; g Center for Interdisciplinary Addiction Research (ZIS), Department of Psychiatry and Psychotherapy, University Medical Center Hamburg-Eppendorf (UKE), Martinistraße 52, 20246 Hamburg, Germany; h Program on Substance Abuse & WHO European Region Collaboration Centre, Public Health Agency of Catalonia, Roc Boronat Street 81 - 95, 08005, Barcelona, Catalonia, Spain

**Keywords:** China, alcohol, attributable mortality, alcohol control policies, economy, anti-corruption campaign

## Abstract

Yearly adult *per capita* consumption of alcohol in China between 2016 and 2019 decreased by 2.4 L of pure alcohol, or 33%. According to the World Health Organization, this decrease in consumption was accompanied by reductions in alcohol-attributable mortality of 23% between 2015 and 2019. This paper examines the contribution of alcohol control policies in China to these public health gains. A systematic search of the literature was conducted on alcohol control policies and their effectiveness in China as part of a larger search of all countries in WHO Western Pacific Region. In addition to articles on empirical evidence on the impact of such alcohol control policies, we also searched for reviews. The plausibility of changes of traditional alcohol control policies (taxation increases, availability restrictions, restriction on advertisement and marketing, drink-driving laws, screening and brief interventions) in explaining reductions of consumption levels and attributable mortality rates was explored. There was some progress in the successful implementation of strict drink-driving policies, which could explain reductions in traffic injuries, including fatalities. Other traditional alcohol control policies seem to have played a minimal role in reducing alcohol consumption and attributable harms during the time period 2016–2019. However, an anti-corruption campaign was extensive enough to have substantially contributed to these reductions. The campaign prohibited the consumption of alcoholic beverages in everyday life of government officials and thus contributed to a de-normalization of alcohol. While this anti-corruption campaign was the only policy to potentially explain marked decreases in levels of alcohol consumption and attributable mortality, more detailed research is required to determine exactly how the campaign achieved these decreases.

## Highlights

• China had decreases of alcohol consumption and attributable mortality since 2016.
• Cardiovascular and digestive diseases were the main causes of death responsible.
• None of the classic alcohol control policies seem to be able to explain these decreases.
• Economic explanations also fail to explain the changes.
• A massive anti-corruption campaign denormalized alcohol use and offers a likely explanation.

## Introduction

**Fig. 1:**
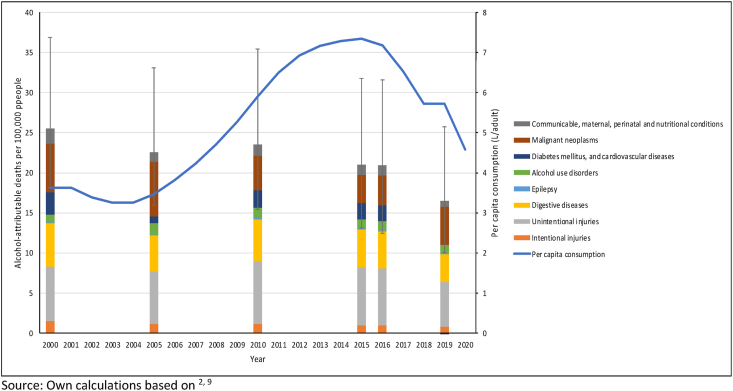
Levels of alcohol consumption and age-standardized alcohol-attributable mortality in China, 2000-2020.

Between 2000 and 2016, levels of alcohol consumption increased steadily in China from 3.89 to 7.42 L of pure alcohol *per capita* (see Fig. 1), in line with a steadily increasing gross domestic product based on purchasing power parity (GDP PPP) *per capita* (Rehm, Babor, Casswell, & Room, 2021). In fact, the correlation between these two indicators over this time period was 0.97 (95% Confidence Interval: 0.92 to 0.99; p = < 0.001; based on the World Health Organization (WHO) (World Health Organization Global Health Observatory, 2024) and World Bank data (The World Bank, 2023a)). Steady increases in alcohol use in the second most populous country in the world have led to predictions that by 2030 the WHO's Western Pacific Region (WPR) will become the region with the highest level of alcohol consumption, and that the modest global goal of reducing alcohol consumption by 10% within 15 years, as specified in the *Global Action Plan For the Prevention and Control of Non-communicable Diseases* (World Health Organization, 2013), will not be reached (Manthey et al., 2019; World Health Organization, 2018). Moreover, numerous calls have been made in high-impact journals for the implementation of strong traditional alcohol control policies aimed at improving the situation (Jiang, Room, & Hao, 2015; Tang et al., 2013).

Since 2016, levels of alcohol consumption in China have decreased. Between 2016 and 2019, consumption decreased by 33%, resulting in a negative association with GDP PPP *per capita* of about the same effect size (−0.99; 95% CI: −0.9996 to −0.3617; p = 0.0184). Such decreases continued after the beginning of the COVID pandemic. It is worth noting that most of the alcohol consumed in China is consumed by males: WHO estimated that 85.3% of all alcohol in China in 2019 was consumed by males, compared to 80% globally (World Health Organization, 2024).

Rates of age-standardized alcohol-attributable mortality did not trend in parallel with the consumption level, but this is not unexpected since alcohol-attributable mortality is more impacted by overall trends in mortality (for further elaboration, see (Rehm, Casswell, Manthey, Room, & Shield, 2021)). And overall age-standardized mortality rates decreased markedly following the economic growth that led China to transition from a low-income country in 1998 to a lower middle-income country in 1999 and an upper-middle income country in 2010 (Scribd Inc., 2024); for impact on level of alcohol use see (Rehm et al., 2024).

Thus, age-standardized alcohol-attributable mortality rates decreased by 18.1% from 2000 to 2016, but during the same period, age-standardized all-cause mortality rates decreased by 29.9%. In other words, the increase in alcohol consumption in this time was slowing down the overall mortality gains associated with economic growth. The average annual rate changes in alcohol-attributable mortality may not accurately represent the yearly change, as the variations in *per capita* alcohol consumption, and presumably alcohol-attributable mortality, followed a curvilinear pattern (see Fig. 1).

However, between 2016 and 2019, there had also been a marked decrease in alcohol-attributable mortality (23%, with overlapping but correlated confidence intervals; Fig. 1), over and above the 5% decrease in all-cause mortality (World Health Organization, 2024).

What role did alcohol control policies in China play in reducing alcohol consumption and resulting mortality rates in that country? An attempt will be made to answer this question by analyzing reviews and evaluations of alcohol control policies in China which were collected via a systematic search.

## Materials and methods

Before introducing the methods of the current review, the basic methods which were applied by the WHO to derive alcohol consumption and attributable harms data will be outlined; these methods are published in detail in the upcoming WHO report on the progress of attaining target 3.5 of the Sustainable Development Goals (World Health Organization, 2024). Alcohol exposure is monitored by the WHO based on changes in recorded, unrecorded, and tourist consumption (Poznyak et al., 2013). The Chinese data were mainly based on recorded consumption monitored by the Chinese National Bureau of Statistics, based on sales, production, import and export. WHO estimated unrecorded and tourist consumption. The data on total alcohol *per capita* consumption and its components are validated by the respective governments.

Alcohol-attributable burdens are estimated using a comparative risk assessment methodology, whereby alcohol-attributable fractions are utilized to estimate the burden of disease that would be prevented under the counterfactual scenario of everyone being a lifetime abstainer from alcohol (Shield et al., 2020). An overview of these procedures can be found in Appendix 1.

### Systematic search of alcohol control policies in China

A systematic search (for search terms, see Appendix 2) was conducted to identify studies on alcohol control policies in China; this search was part of a larger systematic review project on all countries of the WPR (see PROSPERO registration: CRD42023469271). Medline, Embase, and EconLit were systematically searched, in addition to grey literature and reference lists (with full-text screening) for studies published between January 1, 2000 and October 2, 2023, without language restrictions. The search was updated to include literature up to April 30, 2024, with two more publications added as a result. PRISMA will be used to report the results (Page et al., 2021).

For empirical studies evaluating the effects of alcohol control policies, inclusion criteria included intervention or observational study designs (such as quasi-experimental, including time-series analysis, or repeated cross-sectional), alcohol control policy implementation, and evaluation of outcomes including alcohol consumption, mortality and burden of disease, social harms, economic costs, and knowledge of alcohol-attributable harms. Review articles were extracted separately. Conference abstracts, and reports focusing on countries outside the WPR were excluded. Titles, abstracts, and full texts were screened by two independent reviewers (ASH & AF) and disagreements were resolved by a third reviewer (KDS).

Data from eligible studies were extracted using a standardized template and summarized narratively by each alcohol control policy type.

### Assessing plausibility

The major changes in alcohol-attributable mortality between 2016 and 2019 were observed in the following cause-of-death categories (see Fig. 1 above): CVD which changed from a standardized mortality burden of 2.00 to −0.36 deaths per 100,000, i.e., from a major alcohol-attributable harm to a category where alcohol is net-protective; unintentional injury which changed from 7.08 to 5.62 deaths per 100,000 (with 59% of this change in road-traffic injury); and digestive disease from 4.64 to 3.46 deaths per 100,000. Cancer mortality increased, but this indicator does not change rapidly when changes in consumption level occur (e.g., (Gapstur et al., 2023)), and may, in fact, take decades before such a change is reflected in cancer rates. The other causes of death have been shown to change quickly in response to changes in policy, with most of the effect seen within the first year (for lag structures, see (Holmes, Meier, Booth, Guo, & Brennan, 2012)). We will measure changes in policy against these changes in cause-specific mortality rates.

## Results

For alcohol control policies, 5014 studies were screened after duplicates were removed, and 214 met criteria for full-text review (see Appendix Fig. 1). Of these studies, 12 empirical studies and one review met inclusion criteria that were specific to alcohol control policies in mainland China, four for Hong Kong, and two for Taiwan. With hand searches, we included three more reviews, and the update up to April 2024 added two more empirical studies (Hu et al., 2023; Zhang, Hu, Zhang, & Zheng, 2024); see Appendix 3). For this report, we excluded the reports from Hong Kong and Taiwan, as they had different alcohol control laws and/or different levels of enforcement.

The study designs included interrupted time-series (n = 10; 8 potentially relevant: (Fei et al., 2020; Gu et al., 2021; Hu et al., 2023; Q. Li et al., 2017; J. Liu, Feng, Steel, Zhou, & Astell-Burt, 2023; Xiong, Wang, Xu, & Li, 2019; Xu et al., 2021; Zhao et al., 2016)), repeated cross-sectional (n = 4: (Bhalla et al., 2013; Wang et al., 2015; Xiao, Ning, Schwebel, & Hu, 2017)), one longitudinal panel (Zhang et al., 2024) and one review (Hu et al., 2022)). In addition, using hand searches, we included three more reviews (for a listing of all studies, see Appendix 3; for a short description of each study see Appendix 4) (Guo & Huang, 2015; Ji, Bai, Xu, Liu, & Jia, 2021; S. Liu et al., 2022). The majority of the evaluation studies were sub-national (n = 7).

What were the potential contributions of various alcohol control policies to the drop in levels of consumption and attributable harms? For categorization, the classification of interventions by the WHO as specified in the SAFER initiative were followed (World Health Organization, 2022), which included the three best buys—increasing prices via taxation, reducing availability of alcoholic beverages, banning of marketing and advertisement (Chisholm et al., 2018)—and the use of drink-driving countermeasures, screening, and brief interventions (Rekve et al., 2019).

### Potential contribution of alcohol taxation laws to reducing affordability

Based on the last tax survey from the WHO for the year 2022, the tax shares for the most-consumed alcoholic beverages in China were 2.12%, and 16.30%, for beer and Chinese spirits, respectively (World Health Organization, 2023a). “Tax share” denotes the proportion of the retail price collected by the government as revenue (World Health Organization, 2023b). While these tax-share values suggest that tax increases would result in public health benefits (Hu et al., 2023), current taxation rates in China are too low. The only fiscal change since 2016, an adjustment of the minimum taxable price will be adjusted from 50–70%–60% seems unlikely to contribute to the marked decreases in alcohol consumption and attributable harms since 2016 in China (for a more detailed discussion of past policies, see (Guo & Huang, 2015; Hu et al., 2022)).

### Potential contribution of availability restrictions

Several traditional pillars of availability restrictions exist, but these are often not well enforced in China (Guo & Huang, 2015; Ji et al., 2021; S. Liu et al., 2022). Consequently, the availability of alcohol is subject to licensing covering production, quality control, regulation of distribution, and other business activities (S. Liu et al., 2022). However, China has no enforceable legal provision which regulates when or where alcoholic beverages may be sold (Ji et al., 2021). Indeed, online delivery has become an important tool for the supply of alcohol in China, and it is not subject to any regulation. Also, policies on alcohol distribution which are currently in place are poorly implemented, and policies regarding management of the supply of alcohol and of drinking environments are not implemented (S. Liu et al., 2022).

A potentially effective policy is to prevent access to alcohol among younger people (Babor et al., 2023). All reviews indicate that existing regulations for the protection of minors are not well enforced (Guo & Huang, 2015; Hu et al., 2022; Ji et al., 2021; S. Liu et al., 2022). For instance, a survey done in 2021 across seven provinces in China indicated that 82.5% of middle and high school students who had attempted to purchase alcohol in the past 30 days had not been refused based on being under age (Chinese Center for Disease Control and Prevention, 2021).

However, there is one non-traditional policy intervention which restricts availability and may have been a large contributor to the reduction of alcohol consumption and attributable harms. Before 2012, it was not unusual for government officials, civil and military included, to drink alcohol during working hours at government expense. This was justified by deep-rooted beliefs that “friendship can be measured by how much you drink” or “drinking is essential in business affairs” (Tang et al., 2013). However, holding lavish banquets as a part of work to foster intragovernmental relations or interactions with the private sector was also seen as potentially corrupt behaviour (Hu et al., 2022; Shu & Cai, 2017). To address this practice, an “anti-corruption” regulation has existed since 2012, including a ban on drinking by military personnel and the “eight rules" forbidding government officials from drinking alcohol while working (Hu et al., 2022). The implementation of this anti-corruption campaign in China was followed by declines in the sale of luxury alcohol products and declines in drinking among government officials, as evidenced by a marked decrease in the proportion of government officials who engaged in the consumption of *baijiu* (a clear grain alcohol) (Insight and Info, 2018). Since shrinking *baijiu* consumption was the main factor in the reduction of overall levels of consumption in China (Hu et al., 2022), it can be surmized that decreases in *baijiu* consumption were driven primarily by the anti-corruption campaign.

### Potential contribution of policies to restrict advertisement and marketing

Regulation of the marketing of alcohol is the third of the WHO's “best buy” policies. Alcohol marketing and advertisement is controlled, in part, by regulations affecting advertising content, time, frequency, and protection of minors (S. Liu et al., 2022). However, there is no comprehensive marketing ban in place, and there are no regulations affecting new forms of alcohol marketing, such as digital marketing, including the promotion of the online delivery of alcohol. As the immediate impact of bans on marketing is limited (Manthey, Jacobsen, Klinger, Schulte, & Rehm, 2024), and as marketing seems to be a measure designed primarily to de-normalize alcohol use in the long term, it appears that marketing laws were not the cause of the abrupt and massive changes in alcohol consumption levels and attributable harms.

### Potential contribution of drink-driving legislation

Most of the published evaluations of alcohol policies addressed the implementation of more severe penalties for drink-driving offences (Franklin, 2023 (in preparation)). According to the most recent comprehensive evaluation of national data from mainland China (J. Liu et al., 2023), following the 2011 amendment of the Criminal Law and Road Traffic Law designating drink-driving as a criminal offence (Y. Li, Xie, Nie, & Zhang, 2012), the immediate daily mortality rate for road traffic injuries dropped by 1.57% (RR = 0.9843, 95% CI: 0.9444 to 1.0259), while the slope significantly decreased by 0.04% (RR = 0.9996, 95% CI: 0.9994 to 0.9997) compared with the period before this law was amended. Even though road traffic fatalities were the 10th leading cause of death in China in 2019 (The Institute for Health Metrics and Evaluation (IHME), 2023), and alcohol-attributable traffic fatalities were reduced by 13,000 between 2016 and 2019, this comprised 20% of all net alcohol-attributable deaths ((World Health Organization, 2024); see also (Gu et al., 2021) and the composition of causes of death reduced above), and thus cannot be responsible for the overall changes in alcohol-attributable mortality.

However, a recent evaluation of alcohol intake before and after the 2011 change in the law indicated that alcohol-intake declines were also associated with the introduction of drink-driving as a criminal offence (Zhang et al., 2024). However, the changes could not have impacted on overall adult alcohol *per capita* consumption at the time since levels of alcohol consumption in 2011 continued to increase. Thus, it may be concluded that drink-driving laws have had a limited impact on overall levels of consumption.

In summary, while drink-driving legislation certainly contributed to the decrease in alcohol-attributable harms in China, it is unlikely to have been the major driver of the 2016–2019 reversal, which was mainly led by changes in cardiovascular and digestive disease mortality.

### Potential contribution of screening and brief interventions

The last policy of the SAFER interventions involves screening for hazardous consumption, followed by treatment or brief interventions based on levels of detected consumption and alcohol problems (Babor et al., 2023). While individual-level studies point to some effectiveness of screening and brief interventions in China, (e.g., (Zhai et al., 2022)) to date, except for modelling studies (Rehm, Shield, Gmel, Rehm, & Frick, 2013), there is no evidence regarding the population-level effectiveness of this policy in reducing levels of alcohol use. Moreover, current screening rates in China seem too low to predict such population-level changes (Paschall et al., 2022).

## Discussion and conclusion

Levels of alcohol consumption and attributable harms have decreased markedly in China since 2016. While data up to and including 2019 are presented herein, there are indications that such reductions have continued in China, triggered in part by COVID-19 measures (World Health Organization, 2024). The classic tools of alcohol control policies (in particular, taxation increases, restrictions of temporal availability via trading hours for on- and off-premise alcohol consumption, bans on marketing; see (Rehm, Lange, et al., 2023)) do not appear to have played a large role in the observed 2016–2019 decreases.

The anti-corruption campaign of the Chinese government offers a potential explanation for a large part of the consumption and harms reductions. Unfortunately, and this is the most notable limitation of this paper, there is insufficient research using modern techniques to ascertain a causal effect on the population impacts of the anti-corruption campaign on alcohol-attributable mortality (Shadish, Cook, & Campbell, 2002). Overall, current evidence does not allow for a clear causal interpretation. However, the limited role of the classic alcohol control policies seems to be clear here—given the observed minimal changes made to these policies in China over the time period which could have affected alcohol use and mortality (Babor et al., 2023).

How plausible are the conclusions reached in this paper? First, the reliability of the underlying information about changes in consumption levels and attributable harms must be further analyzed, then alternative explanations for the decreases should be investigated, and, finally, the effects of the anti-corruption campaign on consumption levels and attributable harms must be examined. Alcohol consumption statistics are considered to be quite reliable, as they are based primarily on recorded consumption (in China, recorded consumption in 2020 constituted more than 80% of total consumption (World Health Organization Global Health Observatory, 2024) which is, in turn, based on taxation or production, and import and export statistics (Poznyak et al., 2013; Rehm, Klotsche, & Patra, 2007). These aggregate statistics have much less bias than survey data (Gmel & Rehm, 2004) based on self-report and usually incomplete sampling frames. However, it should be noted that all data sources from China point towards reductions in alcohol consumption, i.e., for all major alcoholic beverages (baijiu, wine and beer). This holds true even for surveys.

Alcohol-attributable mortality is based on combining alcohol exposure (Rehm et al., 2010) with relative risk curves (Rehm, Rovira, Llamosas-Falcón, & Shield, 2021) derived from meta-analyses (Rehm et al., 2017) to derive attributable fractions, which are then applied to mortality statistics (Ezzati, Lopez, Rodgers, & Murray, 2004). This methodology is standard for comparative risk analyses, irrespective of whether they have been conducted by the Global Burden of Disease Group or by the WHO, but many assumptions have been made (Greenland, 2015). Most critical for the current exercise is the assumption that the globally derived risk relations are correct for China. There are serious doubts about two main disease categories: for cancer, the risk relations are an underestimate, as they do not take into account the increased relative risk for cancer in the Chinese population. This increased risk is due to the relatively high prevalence of a genetic constellation in a sizable number of people of Chinese ancestry which leads to a slower metabolism in the breakdown of acetaldehyde, a known carcinogen (International Agency for Research on Cancer, 2010). These individuals clearly are at higher risk, and thus should show higher attributable fractions than the global average (Roerecke et al., 2015). The second doubt arises from the protective effects of light to moderate alcohol consumption on ischemic disease (i.e., ischemic heart disease and ischemic stroke), which seem to be overestimated for China given the last epidemiological results, especially when considering the genetically informed results (Im et al., 2023; Millwood et al., 2019; Millwood et al., 2023). While deaths caused by cardiovascular diseases constitute the largest contributor to the decline in mortality between 2016 and 2019, the same assumptions were used for all morality rates in Fig. 1, and it is unclear how these biases could potentially explain the changes in mortality after 2016.

One classic explanation for the observed changes in consumption and mortality outside of alcohol control policies is the impact of the improving economy on the general population. During the trajectory towards becoming high-income economies, the increases in *per capita* alcohol use in lower-income countries is usually the greatest in the earlier stages, i.e., when the GDP PPP *per capita* increases during the low-income phase or the lower middle-income phase (Shield, Rehm, Patra, Sornpaisarn, & Rehm, 2011). Thus, the current wealth of China would not typically lead to further increases in alcohol *per capita* consumption. Moreover, economic growth in China slowed in recent years (average annual percentage increase 2000–2016: 10.04%; in 2016–2020: 6.29%), but average disposable income increased in the last years (National Bureau of Statistics of China, 2022), outpacing inflation (The World Bank, 2023b) annually by an average of 0.75% from 2000 to 2016 and 2.94% from 2016 to 2020. The growth outlook for China is expected to decline from 5.4% in 2023 to 4.6% in 2024 and to 3.5% by 2028 as a result of several external factors, including elevated debt and a housing-market crisis (International Monetary Fund, 2023; The World Bank, 2023c).

The outlook of Chinese citizens may have changed as a result of recent economic changes. However, neither economic reasons nor personal perceptions tend to change so abruptly that they would explain the relatively rapid changes in alcohol consumption and attributable harms starting in 2016, although both factors may still have played a role.

One reviewer cited the decrease in alcoholic strength, especially in beer products after the introduction of low- and no alcohol products, as a potential explanation for the decrease. This explanation does not seem possible, however. First, even in markets with the largest market share of low- and no-alcohol products, the overall substitution of higher strength was limited, and thus public health effects were very small (Rehm, Rovira, Manthey, & Anderson, 2023). Second, market data on this segment in China indicate that such a large effect on consumption and harm is impossible (IWSR, 2023).

Has the anti-corruption campaign been far-reaching enough to explain such a change? As of 2023, approximately 2–3 million government officials have been prosecuted as a result of this campaign, so the campaign appears to have been quite widespread (Jin, 2023). Overall, according to data from the International Labour Organization for 2012, approximately 28% of the overall workforce in China works in the public service (International Labour Organization (ILOSTAT), 2024). This proportion seems large enough to explain even the large reductions of consumption as seen (see also (Shu & Cai, 2017)). It should be noted that the public service includes not only the federal government, but also local governments and state-owned or state-run enterprises (Jin, 2023).

Overall, while the anti-corruption campaign was not focused on alcohol control, it may have inadvertently resulted in an effective reduction in alcohol use and subsequent alcohol-attributable mortality. However, it is necessary to note that alcohol control played only a part in this campaign. Additional evidence is needed to quantify the contribution of the anti-corruption campaign to the decreases seen in alcohol consumption and attributable harms.

## Funding

This research was financially supported, in part, by contributions of the WHO Western Pacific Region (WPRO-2023-08/DHP_PND/210851) and of WHO Headquarters to the WHO Collaboration Centre in Toronto (2023/1420956-0). JR and ASH also were supported by the Canadian Institutes of Health Research (FRN 477887).

## CRediT authorship contribution statement

**Jürgen Rehm:** Writing – review & editing, Writing – original draft, Methodology, Investigation, Funding acquisition, Data curation, Conceptualization. **Kevin Shield:** Writing – review & editing, Resources, Methodology, Formal analysis. **Ahmed S. Hassan:** Writing – review & editing, Data curation. **Ari Franklin:** Writing – review & editing, Data curation, Conceptualization.

## Footnotes

Appendix A Supplementary data to this article can be found online at https://doi.org/10.1016/j.alcohol.2024.07.002.
