# Supplementary material for: The role of alcohol control policies in the reversal of alcohol consumption levels and resulting attributable harms in China
Source: Alcohol. 2024 Dec;121:19–25. doi: 10.1016/j.alcohol.2024.07.002 (PMC11633449; doi:10.1016/j.alcohol.2024.07.002)
Supplement: Multimedia component 1 [file mmc1.docx]

# Appendices of

# The role of alcohol control policies in the reversal of alcohol consumption levels and resulting attributable harms in China

Jürgen Rehm^a-h^, Kevin Shield^a-d^, Ahmed S. Hassan^a,d^, Ari Franklin^a^

1. Institute for Mental Health Policy Research, Centre for Addiction and Mental Health, 33 Ursula Franklin Street, Toronto, Ontario, Canada, M5S 2S1
2. Campbell Family Mental Health Research Institute, Centre for Addiction and Mental Health, 33 Ursula Franklin Street, Toronto, Ontario, Canada, M5S 2S1
3. PAHO/WHO Collaborating Centre at CAMH, Toronto, Canada & WHO European Region Collaborating Centre at the Public Health Institute of Catalonia, Roc Boronat Street 81 - 95, 08005, Barcelona, Catalonia, Spain
4. Dalla Lana School of Public Health, University of Toronto, 155 College Street, 6th Floor, Toronto, Ontario, Canada, M5T 3M7
5. Department of Psychiatry, Faculty of Medicine, University of Toronto, 250 College Street, 8^th^ floor, Toronto, Ontario, Canada, M5T 1R8
6. Faculty of Medicine, Institute of Medical Science, University of Toronto, Medical Sciences Building, 1 King’s College Circle, Room 2374, Toronto, Ontario, Canada, M5S 1A8
7. Center for Interdisciplinary Addiction Research (ZIS), Department of Psychiatry and Psychotherapy, University Medical Center Hamburg-Eppendorf (UKE), Martinistraße 52, 20246 Hamburg, Germany
8. Program on Substance Abuse & WHO European Region Collaboration Centre, Public Health Agency of Catalonia, Roc Boronat Street 81 - 95, 08005, Barcelona, Catalonia, Spain

## Appendix 1: A short description of the underlying comparative risk assessment

### Estimation of the alcohol-attributable burden of disease

A comparative risk assessment framework (CRA;^1^; for alcohol: ^2^) was employed to assess the alcohol-attributable burden of disease in 2019. More specifically, estimates were made using a Levin-based population-attributable fraction (PAF) method integrating alcohol exposure data with corresponding relative risks (RRs;^3^). As is customary for CRAs on alcohol, lifetime abstention from alcohol was used to establish the theoretical minimum risk exposure level based on historical precedent and evidence that lifetime abstinence from alcohol may result in the lowest risk of overall health loss.^2^

The following disease conditions were selected as being caused by alcohol by the WHO Technical Advisory Group on Alcohol and Drug Epidemiology (for details, including ICD codes, see ^4^): infectious disease: TB, HIV/AIDS and other sexually transmitted disease, pneumonia; cancer: mouth & oropharyngeal, liver, colorectal, female breast; neuropsychiatric disease; diabetes; cardiovascular disease: hypertensive disease, ischemic heart disease, cardiomyopathy, atrial fibrillation and flutter, ischemic stroke, hemorrhagic stroke; gastrointestinal disease: liver cirrhosis, pancreatitis; unintentional injury: traffic, drowning, fall, poisoning, other; intentional injury: self-inflicted injury, homicide, others.

RR estimates used to assess the alcohol-attributable burden of disease were obtained from meta-analyses, the selection of which was also determined by the WHO Technical Advisory Group (for details, see RR functions below). In order for an RR estimate to be selected, the source had to meet the selection criteria of having outcomes that matched WHO-reported disease and injury categories, used lifetime abstainers as the reference group, and modelled the continuous dose-response association between alcohol and cancer while controlling for confounders.

Indicators for alcohol-attributable harm included deaths, years lived with disability (YLD), disability-adjusted life years (DALYs) lost, and years of life lost (YLL; for definitions, see ^5^ which were acquired from the WHO’s Global Health Estimates.^6^ Population data were obtained from the United Nations Population Division.^7^ For comparability with WHO Global Status Reports, age-standardized rates of mortality and morbidity per 100,000 people were calculated using the WHO standard.^8^

### Uncertainty estimates for burden

A Monte-Carlo approach was used to measure the 95% uncertainty intervals (UIs) for the estimates of the alcohol-attributable burden of disease.^9^ Employing a simulation creating 1,000 estimates, the 2·5^th^ and 97·5^th^ percentiles were constructed for the estimates of alcohol-attributable harms.

### Relative Risks for partially alcohol-attributable disease categories

| Disease | Sex / Age | Relative risk function (where x is the daily alcohol consumption in g) | Source |
| --- | --- | --- | --- |
| Tuberculosis (ICD-10 coding: A15-19, B90) | Both / All ages | RR_CD_ = exp(x∙β1)  β1 = 0·0179695 | Imtiaz et al·, 2017 ^10^ |
| HIV/AIDS (ICD-10 coding: B20-24) | Males / All ages | if(x ≤ 61) RR_CD_ = 1  if(61 > x) RR_CD_ = β1  β1 = ln(1.54) | Rehm et al., 2017 ^11^ |
|  | Females / All ages | if(x ≤ 49) RR_CD_ = 1  if(49 > x) RR_CD_ = β1  β1 = ln(1.54) | Rehm et al., 2017 ^11^ |
| Lower respiratory infections (ICD-10 coding: J09-22, P23, U04) | Both / All ages | RR = exp(β1∙( (x + 0·0399999618530273) / 100) )  β1 = 0·4764038 | Samokhvalov et al., 2010 ^12^ |
| Lip and oral cavity cancer  (ICD-10 codes: C00-08) | Both / All ages | RR_CD_ = exp(x∙β1 + x^2^∙β2)  β1 = 0.02474  β2 = -0·00004 | Bagnardi et al·, 2015 ^13^ |
| Other pharyngeal cancers  (ICD-10 codes: C09-10,  C12-14) | Both / All ages | RR_CD_ = exp(x∙β1 + x^2^∙β2)  β1 = 0·02474  β2 = -0·00004 | Bagnardi et al·, 2015 ^13^ |
| Oesophagus cancer  (ICD-10 codes: C15) | Both / All ages | RR_CD_ = exp(x∙β1 + x^2^∙β2)  β1 = 0·0132063596418668  β2 = -4·14801974664481*10^-08^ | Bagnardi et al·, 2015 ^13^ |
| Colon and rectum cancers  (ICD-10 codes: C18-21) | Both / All ages | RR_CD_ = exp(x∙β1)  β1 = 0·006765865 | Vieira et al·, 2017 ^14^ |
| Liver cancer  (ICD-10 codes: C22) | Both / All ages | RR_CD_ = exp(x∙β1)  β1 = 0·003922071 | World Cancer Research Fund International, 2018 ^15^ |
| Larynx cancer  (ICD-10 codes: C32) | Both / All ages | RR_CD_ = exp(x∙β1 + x^2^∙β2)  β1 = 0·01462  β2 = -0·00002 | Bagnardi et al·, 2015 ^13^ |
| Breast cancer  (ICD-10 codes: C50) | Females / All ages | RR_CD_ = exp(x∙β1)  β1 = 0·018232156 | Sun, 2020 ^16^ |
| Cervix uteri cancer  (ICD-10 codes: C53) | Females / All ages | if(x ≤ 49) RR_CD_ = 1  if(49 > x) RR_CD_ = β1  β1 = ln(1·54) | Rehm et al·, 2017 ^11^ |
| Diabetes mellitus (ICD-10 coding: E10–E14 (minus E10·2–E10·29, E11·2–E11·29, E12·2, E13·2–E13·29, E14·2)) | Male / All ages | RR_CD_ = exp(x∙β1)  β1 = ln(1·18) | World Health Organization 2023 ^17^ |
|  | Females / All ages | RR_CD_ = exp( x∙β1+  β2 ∙ ( pmax((x - 1·000)/12·9940517165868, 0)^3 + ((20·815 - 1) ∙ pmax((x - 47·840)/12·9940517165868, 0)^3 - (47·840 - 1) ∙ (pmax((x - 20·815)/12·9940517165868, 0)^3)) / (47·840 - 20·815) ) +  β3*( pmax((x - 9·065)/12·9940517165868, 0)^3 + ((20·815 - 9·065) ∙ pmax((x - 47·840)/12·9940517165868, 0)^3 - (47·840 - 9·065) ∙ (pmax((x - 20·815)/12·9940517165868, 0)^3)) / (47·840 - 20·815) )  β1 = -0·03892253  β2 = 0·20524216  β3 = -0·34804082 | World Health Organization 2023 ^17^ |
| Epilepsy (ICD-10 coding: G40-41) | Both / All ages | RR_CD_ = exp(β1∙x + 0·5100)  β1 = 1·22861 | Samokhvalov et al·, 2010 ^18^ |
| Hypertensive heart disease (ICD-10 coding: I10-15) | Male / All ages | If(0 ≤ x <10) RR_CD_ = exp(β1∙x)  If(10 ≤ x <30) RR_CD_ = exp(β1*10 + β2∙ (x-10) )  If(30 ≤ x) RR_CD_ = exp(β1*10 + β2*(20) + β3*(x-30) )  β1 = 0·013976194  β2 = 0·00689349  β3 =0·002942025 | Liu et al·, 2020 ^19^ |
|  | Females / All ages | If(0 ≤ x <10) RR_CD_ = exp(β1*x)  If(10 ≤ x <30) RR_CD_ = exp(β1*10 + β2*(x-10) )  If(30 ≤ x) RR_CD_ = exp(β1*10 + β2*(20) + β3*(x-30) )  β1 = 0·005826891  β2 = 0·005362277  β3 =0·005605865 | Liu et al·, 2020 ^19^ |
| Ischaemic heart disease (ICD-10 coding: I20-25) | Males /  15 to 34 years of age | $\mathrm{If}\left( x<60 \right)\mathrm{RR}_{\mathrm{CD}}=exp(\beta_{1}\cdot\left( \beta_{2}\cdot\sqrt{y_{1}}+\beta_{3}\cdot y_{1}^{3} \right))$  $\mathrm{If}\left( 60\leq x<100 \right)\mathrm{RR}_{\mathrm{CD}}=0\cdot04571551+exp(\beta_{1}\cdot\left( \beta_{2}\cdot\sqrt{y_{2}}+\beta_{3}\cdot y_{2}^{3} \right))$  $\mathrm{If}\left( 100\leq x \right)\mathrm{RR}_{\mathrm{CD}}=exp(\beta_{4}\cdot\left( x-100) \right)-1+0\cdot04571551+exp(\beta_{1}\cdot\left( \beta_{2}\cdot\sqrt{y_{2}}+\beta_{3}\cdot y_{2}^{3} \right))$  Where:$y_{1}=\frac{x+0\cdot0099999997764826}{100}$  $y_{2}=\frac{60+0\cdot0099999997764826}{100}$  β1 = 1·111874  β2 = -0·4870068  β3 = 1·550984  β4 = 0·012 | Rehm et al., 2016 ^20^ based on Roerecke & Rehm, 2012 ^21^ |
|  | Males /  35 to 64 years of age | $\mathrm{If}\left( x<60 \right)\mathrm{RR}_{\mathrm{CD}}=exp(\beta_{1}\cdot\left( \beta_{2}\cdot\sqrt{y_{1}}+\beta_{3}\cdot y_{1}^{3} \right))$  $\mathrm{If}\left( 60\leq x<100 \right)\mathrm{RR}_{\mathrm{CD}}=0\cdot04571551+exp(\beta_{1}\cdot\left( \beta_{2}\cdot\sqrt{y_{2}}+\beta_{3}\cdot y_{2}^{3} \right))$  $\mathrm{If}\left( 100\leq x \right)\mathrm{RR}_{\mathrm{CD}}=exp(\beta_{4}\cdot\left( x-100) \right)-1+0\cdot04571551+exp(\beta_{1}\cdot\left( \beta_{2}\cdot\sqrt{y_{2}}+\beta_{3}\cdot y_{2}^{3} \right))$  Where:$y_{1}=\frac{x+0\cdot0099999997764826}{100}$  $y_{2}=\frac{60+0\cdot0099999997764826}{100}$  β1 = 0·757104  β2 = -0·4870068  β3 = 1·550984  β4 = 0·012 | Rehm et al·, 2016 ^20^ based on Roerecke & Rehm, 2012 ^21^ |
|  | Males /  65 years of age and older | $\mathrm{If}\left( x<60 \right)\mathrm{RR}_{\mathrm{CD}}=\exp\left( \beta_{1}\cdot\left( \beta_{2}\cdot\sqrt{y_{1}}+\beta_{3}\cdot y_{1}^{3} \right) \right)$  $\mathrm{If}\left( 60\leq x<100 \right)\mathrm{RR}_{\mathrm{CD}}=0\cdot04571551+exp(\beta_{1}\cdot\left( \beta_{2}\cdot\sqrt{y_{2}}+\beta_{3}\cdot y_{2}^{3} \right))$  $\mathrm{If}\left( 100\leq x \right)\mathrm{RR}_{\mathrm{CD}}=exp(\beta_{4}\cdot\left( x-100) \right)-1+0\cdot04571551+exp(\beta_{1}\cdot\left( \beta_{2}\cdot\sqrt{y_{2}}+\beta_{3}\cdot y_{2}^{3} \right))$  Where $y_{1}=\frac{x+0\cdot0099999997764826}{100}$  $y_{2}=\frac{60+0\cdot0099999997764826}{100}$  β1 = 1·035623  β2 = -0·4870068  β3 = 1·550984  β4 = 0·012 | Rehm et al·, 2016 ^20^ based on Roerecke & Rehm, 2012 ^21^ |
|  | Females /  15 to 34 years of age | $\mathrm{If}\left( x<30\cdot3814 \right)\mathrm{RR}_{\mathrm{CD}}=exp(\beta_{1}\cdot\left( \beta_{2}\cdot y_{1}+\beta_{3}\cdot y_{1}\cdot ln(y_{2}) \right))$  $\mathrm{If}\left( 30\cdot3814\leq x \right)\mathrm{RR}_{\mathrm{CD}}=exp(\beta_{4}\cdot\left( x-30\cdot3814) \right)-1+\exp\left( \beta_{1}\cdot\left( \beta_{2}\cdot y_{2}+\beta_{3}\cdot y_{2}\cdot ln(y_{2}) \right) \right))$  Where:$y_{1}=\frac{x+0\cdot0099999997764826}{100}$  $y_{2}=\frac{30\cdot3814+0\cdot0099999997764826}{100}$  β1 = 1·111874  β2 = 1·832441  β3 = 1·538557  β4 = 0·01 | Rehm et al·, 2016 ^20^ based on Roerecke & Rehm, 2012 ^21^ |
|  | Females /  35 to 64 years of age | $\mathrm{If}\left( x<30\cdot3814 \right)\mathrm{RR}_{\mathrm{CD}}=exp(\beta_{1}\cdot\left( \beta_{2}\cdot y_{1}+\beta_{3}\cdot y_{1}\cdot ln(y_{2}) \right))$  $\mathrm{If}\left( 30\cdot3814\leq x \right)\mathrm{RR}_{\mathrm{CD}}=exp(\beta_{4}\cdot\left( x-30\cdot3814) \right)-1+\exp\left( \beta_{1}\cdot\left( \beta_{2}\cdot y_{2}+\beta_{3}\cdot y_{2}\cdot ln(y_{2}) \right) \right))$  Where:$y_{1}=\frac{x+0\cdot0099999997764826}{100}$  $y_{2}=\frac{30\cdot3814+0\cdot0099999997764826}{100}$  β1 = 1·035623  β2 = 1·832441  β3 = 1·538557  β4 = 0·009300 | Rehm et al·, 2016 ^20^ based on Roerecke & Rehm, 2012 ^21^ |
|  | Females /  65 years of age and older | $\mathrm{If}\left( x<30\cdot3814 \right)\mathrm{RR}_{\mathrm{CD}}=exp(\beta_{1}\cdot\left( \beta_{2}\cdot y_{1}+\beta_{3}\cdot y_{1}\cdot ln(y_{2}) \right))$  $\mathrm{If}\left( 30\cdot3814\leq x \right)\mathrm{RR}_{\mathrm{CD}}=exp(\beta_{4}\cdot\left( x-30\cdot3814) \right)-1+\exp\left( \beta_{1}\cdot\left( \beta_{2}\cdot y_{2}+\beta_{3}\cdot y_{2}\cdot ln(y_{2}) \right) \right))$  Where:$y_{1}=\frac{x+0\cdot0099999997764826}{100}$  $y_{2}=\frac{30\cdot3814+0\cdot0099999997764826}{100}$  β1 = 0·757104  β2 = 1·832441  β3 = 1·538557  β4 = 0·006800 | Rehm et al·, 2016 ^20^ based on Roerecke & Rehm, 2012 ^21^ |
| Ischaemic stroke (ICD-10 coding: G45–46·8, I63–63·9, I65–66·9, I67·2–67·848, I69·3–69·4) | Males /  15 to 34 years of age | $\mathrm{If}\left( x\leq1 \right)\mathrm{RR}_{\mathrm{CD}}=1-x\cdot(1-exp\left( \beta_{1}\cdot\left( \beta_{2}\cdot\sqrt{y_{1}}+\beta_{3}\cdot\sqrt{y_{1}}\cdot\ln\left( y_{1} \right) \right) \right))$  $\mathrm{If}\left( x>1 \right)\mathrm{RR}_{\mathrm{CD}}=exp(\beta_{1}\cdot\left( \beta_{2}\cdot\sqrt{y_{2}}+\beta_{3}\cdot\sqrt{y_{2}}\cdot ln(y_{2}) \right))$  Where:$y_{1}=\frac{1+0\cdot0028572082519531}{100}$  $y_{2}=\frac{x+0\cdot0028572082519531}{100}$  β1 = 1·111874  β2 = 0·4030081  β3 = 0·3877538 | Rehm et al·, 2016 ^20^ based on Patra et al·, 2010 ^22^ |
|  | Males /  35 to 64 years of age | $\mathrm{If}\left( x\leq1 \right)\mathrm{RR}_{\mathrm{CD}}=1-x\cdot(1-exp\left( \beta_{1}\cdot\left( \beta_{2}\cdot\sqrt{y_{1}}+\beta_{3}\cdot\sqrt{y_{1}}\cdot\ln\left( y_{1} \right) \right) \right))$  $\mathrm{If}\left( x>1 \right)\mathrm{RR}_{\mathrm{CD}}=exp(\beta_{1}\cdot\left( \beta_{2}\cdot\sqrt{y_{2}}+\beta_{3}\cdot\sqrt{y_{2}}\cdot ln(y_{2}) \right))$  Where $y_{1}=\frac{1+0\cdot0028572082519531}{100}$  $y_{2}=\frac{x+0\cdot0028572082519531}{100}$  β1 = 1·035623  β2 = 0·4030081  β3 = 0·3877538 | Rehm et al·, 2016 ^20^ based on Patra et al·, 2010 ^22^ |
|  | Males /  65 years of age and older | $\mathrm{If}\left( x\leq1 \right)\mathrm{RR}_{\mathrm{CD}}=1-x\cdot(1-exp\left( \beta_{1}\cdot\left( \beta_{2}\cdot\sqrt{y_{1}}+\beta_{3}\cdot\sqrt{y_{1}}\cdot\ln\left( y_{1} \right) \right) \right))$  $\mathrm{If}\left( x>1 \right)\mathrm{RR}_{\mathrm{CD}}=exp(\beta_{1}\cdot\left( \beta_{2}\cdot\sqrt{y_{2}}+\beta_{3}\cdot\sqrt{y_{2}}\cdot ln(y_{2}) \right))$  Where$y_{1}=\frac{1+0\cdot0028572082519531}{100}$  $y_{2}=\frac{x+0\cdot0028572082519531}{100}$  β1 = 0·757104  β2 = 0·4030081  β3 = 0·3877538 | Rehm et al·, 2016 ^20^ based on Patra et al·, 2010 ^22^ |
|  | Females /  15 to 34 years of age | $\mathrm{If}\left( x\leq1 \right)\mathrm{RR}_{\mathrm{CD}}=1-x\cdot(1-exp\left( \beta_{2}\cdot\sqrt{y_{1}}+\beta_{3}\cdot y_{1} \right)$  $\mathrm{If}\left( x>1 \right)\mathrm{RR}_{\mathrm{CD}}=exp(\beta_{1}\cdot\left( \beta_{2}\cdot\sqrt{y_{2}}+\beta_{3}\cdot y_{2} \right))$  Where:$y_{1}=\frac{1+0\cdot0028572082519531}{100}$  $y_{2}=\frac{x+0\cdot0028572082519531}{100}$  β1 = 1·111874  β2 = -2·48768  β3 = 3·7087240 | Rehm et al·, 2016 ^20^ based on Patra et al·, 2010 ^22^ |
|  | Females /  35 to 64 years of age | $\mathrm{If}\left( x\leq1 \right)\mathrm{RR}_{\mathrm{CD}}=1-x\cdot(1-exp\left( \beta_{2}\cdot\sqrt{y_{1}}+\beta_{3}\cdot y_{1} \right)$  $\mathrm{If}\left( x>1 \right)\mathrm{RR}_{\mathrm{CD}}=exp(\beta_{1}\cdot\left( \beta_{2}\cdot\sqrt{y_{2}}+\beta_{3}\cdot y_{2} \right))$  Where:$y_{1}=\frac{1+0\cdot0028572082519531}{100}$  $y_{2}=\frac{x+0\cdot0028572082519531}{100}$  β1 = 1·035623  β2 = -2·48768  β3 = 3·7087240 | Rehm et al·, 2016 ^20^ based on Patra et al·, 2010 ^22^ |
|  | Females /  65 years of age and older | $\mathrm{If}\left( x\leq1 \right)\mathrm{RR}_{\mathrm{CD}}=1-x\cdot(1-exp\left( \beta_{2}\cdot\sqrt{y_{1}}+\beta_{3}\cdot y_{1} \right)$  $\mathrm{If}\left( x>1 \right)\mathrm{RR}_{\mathrm{CD}}=exp(\beta_{1}\cdot\left( \beta_{2}\cdot\sqrt{y_{2}}+\beta_{3}\cdot y_{2} \right))$  Where:$y_{1}=\frac{1+0\cdot0028572082519531}{100}$  $y_{2}=\frac{x+0\cdot0028572082519531}{100}$  β1 = 0·757104  β2 = -2·48768  β3 = 3·7087240 | Rehm et al·, 2016 ^20^ based on Patra et al·, 2010 ^22^ |
| Haemorrhagic stroke (ICD-10 coding: I60–62·9, I67·0–67·1, I69·0–69.298) | Males / All Ages | $\mathrm{If}\left( x\leq1 \right)\mathrm{RR}_{\mathrm{CD}}=1-x\cdot(1-exp\left( \beta_{1}\cdot\frac{(1+0.0028572082519531)}{100} \right)$  $\mathrm{If}\left( x>1 \right)\mathrm{RR}_{\mathrm{CD}}=exp(\beta_{1}\cdot\frac{(1+0.0028572082519531)}{100})$  β1 = 0.6898937 | Larsson et al., 2016 ^23^ |
|  | Females / All Ages | $\mathrm{If}\left( x\leq1 \right)\mathrm{RR}_{\mathrm{CD}}=1-x\cdot(1-exp\left( \beta_{1}\cdot\frac{(1+0.0028572082519531)}{100} \right)$  $\mathrm{If}\left( x>1 \right)\mathrm{RR}_{\mathrm{CD}}=exp(\beta_{1}\cdot\frac{(1+0.0028572082519531)}{100})$  β1 = 1.466406 | Larsson et al., 2016 ^23^ |
| Atrial fibrillation and flutter (ICD-10: I48) | Both / All Ages | RR_CD_ = exp(x∙β1)  β1 = 0·00641342 | Larsson et al·, 2014 ^24^ |
| Cirrhosis (ICD-10 coding: K70, K74) | Males / All Ages | $\mathrm{If}\left( x\leq1 \right)\mathrm{RR}_{\mathrm{CD}}=1+x\cdot(exp\left( \left( \beta_{1}+\beta_{2} \right)\cdot\frac{\left( 1+0\cdot1699981689453125 \right)}{100}-1 \right)$  $\mathrm{If}\left( x>1 \right)\mathrm{RR}_{\mathrm{CD}}=exp\left( \left( \beta_{1}+\beta_{2} \right)\cdot\frac{\left( 1+0\cdot1699981689453125 \right)}{100} \right)$  β1 = 1·687111  β2 = 1·106413 | Roerecke et al·, 2019 ^25^ |
|  | Females / All Ages | $\mathrm{If}\left( x\leq1 \right)\mathrm{RR}_{\mathrm{CD}}=1+x\cdot(exp\left( \left( \beta_{1}+\beta_{2} \right)\cdot\sqrt{\frac{\left( 1+0\cdot1699981689453125 \right)}{100}}-1 \right)$  $\mathrm{If}\left( x>1 \right)\mathrm{RR}_{\mathrm{CD}}=exp\left( \left( \beta_{1}+\beta_{2} \right)\cdot\sqrt{\frac{\left( 1+0\cdot1699981689453125 \right)}{100}} \right)$  β1 = 2·351821  β2 = 0·9002139 | Roerecke et al·, 2019 ^25^ |
| Pancreatitis (ICD-10 coding: K85-86) | Males / All Ages | RR_CD_ = exp(x∙β1)  β1 = 0·0173451 | Samakvalov et al·, 2015 ^26^ |
|  | Females / All Ages | $\mathrm{If}\left( x<3 \right)\mathrm{RR}_{\mathrm{CD}}=exp(\beta_{1}\cdot x)$  $\mathrm{If}\left( 3\leq x<15 \right)\mathrm{RR}_{\mathrm{CD}}=exp(\beta_{1}\cdot x+\beta_{2}\cdot\frac{{(x-3)}^{3}}{{(40-3)}^{2}})$  $\mathrm{If}\left( 15\leq x<40 \right)\mathrm{RR}_{\mathrm{CD}}=exp(\beta_{1}\cdot x+\beta_{2}\cdot\frac{{(x-3)}^{3}-\frac{{(x-15)}^{3}*(40-3)}{(40-15)}}{{(40-3)}^{2}})$  $\mathrm{If}\left( 40\leq x<108 \right)\mathrm{RR}_{\mathrm{CD}}=exp(\beta_{1}\cdot x+\beta_{2}\cdot\frac{{(x-3)}^{3}-\frac{{(x-15)}^{3}*\left( 40-3 \right)-{(x-40)}^{3}*(15-3)}{(40-15)}}{{(40-3)}^{2}})$  $\mathrm{If}\left( x>108 \right)\mathrm{RR}_{\mathrm{CD}}=\exp\left( \beta_{1}\cdot108+\beta_{2}\cdot\frac{\left( 108-3 \right)^{3}-\frac{\left( 108-15 \right)^{3}*\left( 40-3 \right)-\left( 108-40 \right)^{3}*\left( 15-3 \right)}{\left( 40-15 \right)}}{\left( 40-3 \right)^{2}} \right)$  β1 = -0·0272886  β2 = 0·0611466 | Samakvalov et al·, 2015 ^26^ |
| Road injuries (ICD-10 coding: V01-04, V06, V09-80, V87, V89, V99) | Both  (Non-heavy episodic drinkers) / All Ages | $\mathrm{RR}_{\mathrm{CD}}=exp(\beta_{1}\cdot x)$  β1 = 0·00299550897979837 | World Health Organization 2018 ^27^ |
|  | Both  (Heavy episodic drinkers) / All Ages | $\mathrm{RR}_{\mathrm{CD}}=exp(\beta_{1}\cdot x+\beta_{2})$  β1 = 0·00299550897979837  β2 = 0·959350221334602 | World Health Organization 2018 ^27^ |
| Unintentional injuries (ICD-10 coding: V01-X40, X43, X46-59, Y40-86, Y88, Y89 (excluding road injuries)) | Both  (Non-heavy episodic drinkers) / All Ages | $\mathrm{RR}_{\mathrm{CD}}=exp(\beta_{1}\cdot x)$  β1 = 0·00199800266267306 | World Health Organization 2018 ^27^ |
|  | Both  (Heavy episodic drinkers) / All Ages | $\mathrm{RR}_{\mathrm{CD}}=exp(\beta_{1}\cdot x+\beta_{2})$  β1 = 0·00199800266267306  β2 = 0·647103242058538 | World Health Organization 2018 ^27^ |
| Intentional injuries (ICD-10 coding: X60-Y09, Y35-36, Y870, Y871) | Both  (Non-heavy episodic drinkers) / All Ages | $\mathrm{RR}_{\mathrm{CD}}=exp(\beta_{1}\cdot x)$  β1 = 0·00199800266267306 | World Health Organization 2018 ^27^ |
|  | Both  (Heavy episodic drinkers) / All Ages | $\mathrm{RR}_{\mathrm{CD}}=exp(\beta_{1}\cdot x+\beta_{2})$  β1 = 0·00199800266267306  β2 = 0·647103242058538 | World Health Organization 2018 ^27^ |

The pmax function compares the values of the two vectors in pairs and returns a final vector that contains the maximum values from each pair· This function is used in restricted cubic splines

## Appendix 2: Search terms

Searches were completed in 3 databases (Embase and Medline via OVID, and EconLit).

**Embase (via OVID)**

1. exp alcohol consumption/

2. (alcohol* or drink* or drunk).ti,ab,kf,kw.

3. (beer or wine or spirit?).ti,ab,kf,kw.

4. 1 or 2 or 3

5. exp health care policy/ or exp public policy/

6. exp drunken driving/

7. (policy or policies).ti,ab,kf,kw.

8. (tax or pric* or minimum pric* or minimum unit pric*).ti,ab,kf,kw.

9. ((sale* or sell*) and hour*).ti,ab,kf,kw.

10. (restrict* or regulat* or prohibit* or ban).ti,ab,kf,kw.

11. (marketing or advertis* or media or sponsor*).ti,ab,kf,kw.

12. 5 or 6 or 7 or 8 or 9 or 10 or 11

13. (Pacific or ASEAN or Polynesia or Melansia or ((Southeast or East) and Asia) or Oceania or Australia or Brunei or Cambodia or China or Cook Islands or Fiji or Japan or Kiribati or Laos or Malaysia or Marshall Islands or Micronesia or Mongolia or Nauru or New Zealand or Niue or Palau or Papua New Guinea or (Philippines or Filipin*) or ((Republic or South) and Korea) or Samoa or Singapore or Solomon Islands or Tonga or Tuvalu or Vanuatu or Viet*).ti,ab,kf,kw.

14. 4 and 12 and 13

15. ("taxonomy" or "syntax" or "excision" or "taxonomic" or "taxonomically" or "taxane" or "taxi" or "taxonic" or parasit* or microbial or phenotyp*).ti,ab.

16. exp animal model/ or exp animal tissue/

17. 14 not (15 or 16)

18. limit 17 to conference abstract

19. 17 not 18

20. limit 19 to yr="2000 -Current"

**Medline (via OVID)**

1. exp Alcohol Drinking/ or exp Alcohol Intoxication/

2. (alcohol* or drink* or drunk).ti,ab,kf,kw.

3. (beer or wine or spirit?).ti,ab,kf,kw.

4. 1 or 2 or 3

5. exp Public Policy/ or exp Health Policy/

6. exp Accidents, Traffic/ or exp driving under the influence/

7. (policy or policies).ti,ab,kf,kw.

8. (tax or pric* or minimum pric* or minimum unit pric*).ti,ab,kf,kw.

9. ((sale* or sell*) and hour*).ti,ab,kf,kw.

10. (restrict* or regulat* or prohibit* or ban).ti,ab,kf,kw.

11. (marketing or advertis* or media or sponsor*).ti,ab,kf,kw.

12. 5 or 6 or 7 or 8 or 9 or 10 or 11

13. (Pacific or ASEAN or Polynesia or Melansia or ((Southeast or East) and Asia) or Oceania or Australia or Brunei or Cambodia or China or Cook Islands or Fiji or Japan or Kiribati or Laos or Malaysia or Marshall Islands or Micronesia or Mongolia or Nauru or New Zealand or Niue or Palau or Papua New Guinea or (Philippines or Filipin*) or ((Republic or South) and Korea) or Samoa or Singapore or Solomon Islands or Tonga or Tuvalu or Vanuatu or Viet*).ti,ab,kf,kw.

14. 4 and 12 and 13

15. limit 14 to yr="2000 -Current"

**Econlit**

S1 ( SU "Food; Beverages; Cosmetics; Tobacco; Wine and Spirits" ) OR ( (alcohol* or drink* or drunk).ti,ab. ) OR ( (beer or wine OR spirit?).ti,ab. )

S2 SU "Health: Government Policy; Regulation; Public Health" OR SU "Marketing" OR KW " Excise Tax" OR KW " Elasticity" OR ( (policy or policies).ti,ab. ) OR ( (tax or pric* or minimum pric* OR minimum unit pric*).ti,ab. ) OR ( ((sale* or sell*) AND hour*).ti,ab. ) OR ( (Restrict* or regulat* or prohibit* or ban).ti,ab,. ) OR ( (Marketing or advertis* or media or sponsor*).ti,ab. )

S3 (Pacific OR ASEAN OR Polynesia OR Melansia or ((Southeast OR East) AND Asia) OR Oceania OR Australia OR Brunei OR Cambodia OR China OR Cook Islands OR Fiji OR Japan OR Kiribati OR Laos OR Malaysia OR Marshall Islands OR Micronesia OR Mongolia OR Nauru OR New Zealand OR Niue OR Palau OR Papua New Guinea OR (Philippines OR Filipin*) OR ((Republic OR South) AND Korea) OR Samoa OR Singapore OR Solomon Islands OR Tonga OR Tuvalu OR Vanuatu OR Viet*)

## Appendix 3: References for China from the systematic search

**Mainland China (excluding Hong Kong)**

1. Bhalla K, Li Q, Duan L, Wang Y, Bishai D, Hyder AA. The prevalence of speeding and drunk driving in two cities in China: a mid project evaluation of ongoing road safety interventions. Injury. 2013;44 Suppl 4(0226040, gon):S49-56.
2. Fei G., Li X., Sun Q., Qian Y., Stallones L., Xiang H., et al. Effectiveness of implementing the criminal administrative punishment law of drunk driving in China: An interrupted time series analysis, 2004-2017. Accid Anal Prev. 2020;144
3. Gu J, Fei G, Meng Y, Sun Q, Qian Y, Jiang X, et al. Revised road traffic safety law and years of life lost due to traffic deaths in China, 2002-2019. Accid Anal Prev. 2021;161(acs, 1254476):106344.
4. Hu A, Zhao X, Room R, Hao W, Xiang X, Jiang H. The effects of alcohol tax policies on alcohol consumption and alcohol use disorders in Mainland of China: an interrupted time series analysis from 1961-2019. Am J Drug Alcohol Abuse. 2023 49(6):746-755. doi: 10.1080/00952990.2023.2280948. PMID: 38059570.
5. Li Q, He H, Duan L, Wang Y, Bishai DM, Hyder AA. Prevalence of drink driving and speeding in China: a time series analysis from two cities. Public Health. 2017;144S(qi7, 0376507):S15–22.
6. Liu J., Feng X., Steel D., Zhou M., Astell-Burt T. Evaluating the effectiveness of implementing a more severe law on prevention of road traffic injury mortality in mainland China: an interrupted time series study based on national mortality surveillance. Inj Prev J Int Soc Child Adolesc Inj Prev. 2023;29(4):309–19.
7. Wang Z., Zhang Y., Zhou P., Shi J., Wang Y., Liu R., et al. The underestimated drink driving situation and the effects of zero tolerance laws in China. Traffic Inj Prev. 2015;16(5):429–34.
8. Xiao W., Ning P., Schwebel D.C., Hu G. Evaluating the effectiveness of implementing a more severe drunk-driving law in China: Findings from two open access data sources. Int J Environ Res Public Health. 2017;14(8):832.
9. Xiong X., Wang Z., Xu R., Li G. Evaluating the impact of criminalizing drunk driving on years of life lost due to road traffic deaths in one megacity, China. Traffic Inj Prev. 2019;20(4):348–52.
10. Xu X.-H., Dong H., Li L., Yang Z., Lin G.-Z., Ou C.-Q. Time-varying effect of drunk driving regulations on road traffic mortality in Guangzhou, China: an interrupted time-series analysis. BMC Public Health. 2021;21(1):1885.
11. Zhang Z, Hu X, Zhang X, Zheng R. Do tougher drinking policies affect men's smoking behavior - Evidence from China. Soc Sci Med. 2024 348:116875. doi: 10.1016/j.socscimed.2024.116875. PMID: 38613870.
12. Zhao A., Chen R., Qi Y., Chen A., Chen X., Liang Z., et al. Evaluating the Impact of Criminalizing Drunk Driving on Road-Traffic Injuries in Guangzhou, China: A Time-Series Study. J Epidemiol. 2016;26(8):433–9.

**Hong Kong**

1. Chung VCH, Yip BHK, Griffiths SM, Yu ELM, Kim JH, Tam WWS, et al. The impact of cutting alcohol duties on drinking patterns in Hong Kong. Alcohol Alcohol. 2013 Nov 1;48(6):720–8.
2. Kim JH, Wong AH, Goggins WB, Lau J, Griffiths SM. Drink driving in Hong Kong: the competing effects of random breath testing and alcohol tax reductions. Addiction. 2013;108(7):1217–28.
3. Ng C.S., Au M., Leung J.Y.Y., Leung G.M., Tian L., Quan J. The impact of road safety policies in a deregulated alcohol tax environment in Hong Kong: a 15-year time series analysis. Addict Abingdon Engl. 2022;117(8):2191–9.
4. Pun VC, Lin H, Kim JH, Yip BH, Chung VC, Wong MC, et al. Impacts of alcohol duty reductions on cardiovascular mortality among elderly Chinese: a 10-year time series analysis. J Epidemiol Community Health. 2013;67(6):514–8.

**Taiwan**

1. Lin CM, Liao CM, Li CY. A time-series analysis of alcohol tax policy in relation to mortality from alcohol attributed causes in Taiwan. J Community Health. 2011;36:986–91.
2. Lin C, Liao C. Alcohol Tax Policy in Relation to Hospitalization from Alcohol‐Attributed Diseases in Taiwan: A Nationwide Population Analysis of Data from 1996 to 2010. Alcohol Clin Exp Res. 2013;37(9):1544–51.

### **Reviews**

1. Hu A, Jiang H, Dowling R, et al. The transition of alcohol control in China 1990-2019: Impacts and recommendations. Int J Drug Policy 2022; 105: 103698. doi: 10.1016/j.drugpo.2022.103698
2. Guo X, Huang YG. The development of alcohol policy in contemporary China. J Food Drug Anal 2015; 23(1): 19-29. doi: 10.1016/j.jfda.2014.05.002
3. Liu S, Huang F, Zhu X, et al. China's Changing Alcohol Market and Need for an Enhanced Policy Response: A Narrative Review. Int J Environ Res Public Health 2022; 19(10) doi: 10.3390/ijerph19105866
4. Ning J, Yamin B, Jianwei X, Min L, Ainan J. Perspectives: Time to Take Actions to Reduce the Harmful Use of Alcohol in China. Chinese Center for Disease Control and Prevention, 2021; 3(4): 74-7.

## Appendix 4: Summary of empirical studies included in the review

| **Citation** | **Study design** | **Policies examined** | **Implementation date** | **Region** | **Outcome(s)** | **Main results** | **Remarks** |
| --- | --- | --- | --- | --- | --- | --- | --- |
| Bhalla K, Li Q, Duan L, Wang Y, Bishai D, Hyder AA. The prevalence of speeding and drunk driving in two cities in China: a mid project evaluation of ongoing road safety interventions. Injury. 2013;44 Suppl 4(0226040, gon):S49-56. | Repeated roadside surveys | Awareness raising campaigns (for drink driving and speeding), social marketing and increased police enforcement | 2011, 2012 | Suzhou and Dalian, China | Changes in knowledge, attitudes, and practices  Prevalence of drink driving (via random breath testing) and speeding  3) road traffic crashes and injuries | No reduction in prevalence of speeding (except in one site in Dalian where it decreased from 70% to <10%)  Drink driving prevalence decreased from 6.4% to 0.5% in Suzhou and from 1.7% to 0.7% in Dalian  No significant decline in injuries and fatalities from crashes.  Public in both cities held attitudes against drink-driving and speeding | Possible concern of underreporting of crash statistics; possible effect of the 2011 drink driving criminalization law unclear |
| Fei G., Li X., Sun Q., Qian Y., Stallones L., Xiang H., et al. Effectiveness of implementing the criminal administrative punishment law of drunk driving in China: An interrupted time series analysis, 2004-2017. Accid Anal Prev. 2020;144 | Interrupted time series analysis | Criminalization of drink driving and penalties | 2011 | China | Traﬃc crashes, injuries, and mortality | Average annual incidences of crashes, mortality, and injuries decreased  The annual traﬃc fatality rates also decreased from around 0.400 (per 10,000 drivers) in 2004 to 0.047 (per 10,000 drivers) in 2017  **Crashes**: Before: -0.140 (-0.158,-0.121); After 0.134 (0.108,0.160) **Mortality**: Before : -0.052 (-0.058,-0.045) After: 0.047 (0.037,0.057) **Injury**: Before: -0.150 (-0.168,-0.131); After: 0.142 (0.116,0.168) | Study period: 2004-2017 Overall, policy was not very effective for assessed outcomes. ITS results of mortality rate are consistent with those reported in GBD 2017 |
| Gu J, Fei G, Meng Y, Sun Q, Qian Y, Jiang X, et al. Revised road traffic safety law and years of life lost due to traffic deaths in China, 2002-2019. Accid Anal Prev. 2021;161(acs, 1254476):106344. | Interrupted time series analysis | Road Traffic Safety Law | 2011 | China | Years of life lost (YLL) per 100,000 population due to traffic crash mortality | Average YLL decreased from 1133.14 to 848.87 per 100,000 persons Pre-intervention (β [95% CI]: -7.61 (-13.02,-2.20); level change: 205.71 (122.93, 288.49); post-intervention (β [95% CI]: -30.11 (-37.75, -22.46) | Study period: 2002-2019 |
| Li Q, He H, Duan L, Wang Y, Bishai DM, Hyder AA. Prevalence of drink driving and speeding in China: a time series analysis from two cities. Public Health. 2017;144S(qi7, 0376507):S15–22. | Repeated cross-sectional - with time series models | Social media campaigns, advocacy for legislative change and law enforcement training | 2010-2014 | Dalian and Suzhou | Prevalence of drink driving and speeding | Speeding: Prevalence decreased from 31.8% (29.2 to 34.5) to 7.4% (7.0 to 7.9) in Dalian and from 13.5% (11.7 to 15.5) to 6.9% (6.4 to 7.4) in Suzhou.  Drink driving: decreased from 1.7% (1.1 to 2.4) in January 2011 to 0.5% (0.2 to 0.9) in November 2014 in Dalian and from 6.4% (5.4 to 7.4) to 0.5% (0.1 to 2.4) in Suzhou during approximately the same period. | Study period: 2010-2014 Low prevalence in Suzhou may be due to automated speed enforcement cameras throughout the city unlike in Dalian |
| Liu J., Feng X., Steel D., Zhou M., Astell-Burt T. Evaluating the effectiveness of implementing a more severe law on prevention of road traffic injury mortality in mainland China: an interrupted time series study based on national mortality surveillance. Inj Prev J Int Soc Child Adolesc Inj Prev. 2023;29(4):309–19. | Interrupted time series analysis | Criminalisation of drink-driving: 2010 (point penalty), 2011 (criminal and road traffic law), 2013 (point penalty revision) | 2010, 2011, 2013 | various sites in China | Road traffic mortality rates | Overall decreasing mortality trend First intervention: Level change 0.9818 (0.9448 to1.0204); slope change: 1.0002 (1.0001 to 1.0004) Second intervention: Level change 0.9843 (0.9444 to 1.0259); slope change: 0.9996 (0.9994 to 0.9997) Third intervention: Level change 1.0379 (1.0047 to 1.0723); slope change: 1.0001 (1.0000 to 1.0001) | Study period: 2007-2015 |
| Wang Z., Zhang Y., Zhou P., Shi J., Wang Y., Liu R., et al. The underestimated drink driving situation and the effects of zero tolerance laws in China. Traffic Inj Prev. 2015;16(5):429–34. | Retrospective examination of the data from the National Disease Surveillance System | Criminal Law Amendment (2011) and Zero tolerance law (2013) | 2011, 2013 | Shandong, China (5 of 17 randomly selected cities) | Traffic mortality from drink driving | 2011-2012: traffic fatalities decreased by 1.01% (26.61% to 25.26%) 2012-2013: traffic fatalities decreased by 6.81% (from 25.26% to 18.45%) | Study period: 2011-2013 |
| Xiao W., Ning P., Schwebel D.C., Hu G. Evaluating the effectiveness of implementing a more severe drunk-driving law in China: Findings from two open access data sources. Int J Environ Res Public Health. 2017;14(8):832. | Repeated cross-sectional analyses | Criminalization of drink-driving | 2011 | China | Road trafﬁc morbidity (YLDs per 100,000 Persons) and mortality | GBD (Crude) Mortality 1990: 0.7454* (0.7429 to 0.7479) 2015: 0.8674* (0.8648 to 0.8700)  GBD (Crude) YLDs per 100,000 Persons 1990: 0.5029* (0.5018 to 0.5040) 2015: 1.1798* (1.1780 to 1.1817)  Police (crude) Mortality 2006: 1.9376* (1.9018 to 1.9741) 2015: 1.3397* (1.3134 to 1.3665) | Study period: 1990 to 2015 for GBD and 2006-2015 for police data (2010 - year before law introduction - used as a reference year) Large gaps between GBD 2015 and police data for estimates of crashes |
| Xiong X., Wang Z., Xu R., Li G. Evaluating the impact of criminalizing drunk driving on years of life lost due to road traffic deaths in one megacity, China. Traffic Inj Prev. 2019;20(4):348–52. | Interrupted time series analysis | Criminalization of drink-driving | 2011 | Tianjin, China | Years of life lost due to road traffic deaths and risk of traffic deaths (RTD) | 778.1-year (95% CI, 200 – 1,355.1) reduction in monthly YLL  11.1% (1.1 to 21.1%) reduced in RTD | Study period: 2008-2014 Decrease in YLL observed in all ages except ≥65 years |
| Xu X.-H., Dong H., Li L., Yang Z., Lin G.-Z., Ou C.-Q. Time-varying effect of drunk driving regulations on road traffic mortality in Guangzhou, China: an interrupted time-series analysis. BMC Public Health. 2021;21(1):1885. | Interrupted time series analysis | Criminalization of drink-driving | 2011 | Guangzhou, China | Road traffic mortality and RTDs per 1 million population | Average risk reduction of RTDs -9.01 (10.05% to -7.62%);  75.82 (54.06 to 92.04) RTDs per 1 million annually were prevented  Standardized annual RTDs (per 1 million population and 1 million motor vehicles): Before 48.1; After 32.9 | Study period: 2008-2018 |
| Zhao A., Chen R., Qi Y., Chen A., Chen X., Liang Z., et al. Evaluating the Impact of Criminalizing Drunk Driving on Road-Traffic Injuries in Guangzhou, China: A Time-Series Study. J Epidemiol. 2016;26(8):433–9. | Interrupted time series analysis | Criminalization of drink-driving | 2011 | Guangzhou, China | Road traffic injuries | Standardized (per 1 million population and 1 million vehicles) daily RTIs decreased 9.6% (6.5% to 12.8%) standardized daily nighttime RTIs decreased by 13.3% (7.2% to 19.3%), while day RTIs by 6.5% (5.8% to 13.4%) standardized daily alcoholism increased 38.8% (35.1% to 42.4%), and non-trafﬁc injuries increased 3.6% (1.4% to 5.8%) | Study period: 2009-2012 |
| Hu A, Zhao X, Room R, Hao W, Xiang X, Jiang H. The effects of alcohol tax policies on alcohol consumption and alcohol use disorders in Mainland of China: an interrupted time series analysis from 1961-2019. Am J Drug Alcohol Abuse. 2023 49(6):746-755. doi: 10.1080/00952990.2023.2280948. PMID: 38059570. | Interrupted time series analysis | Taxation | 1994, 1998, 2001, 2006, 2009, and 2017 | China | Alcohol consumption  Prevalence of alcohol use disorders and associated YLDs per 100,000 population | **Consumption per capita (difference in trend change before and after intervention)** 1994: 0.057 (-0.122 to 0.235) 1998: -0.434 (-0.612 to -0.256) 2000: 0.270 (0.231 to 0.310) 2006: 0.495 (0.421 to 0.569) 2009: -0.426 (-0.541 to -0.311) 2017: -0.454 (-0.563 to -0.344)  **Prevalence of alcohol use disorder (difference in trend change before and after intervention)** 1994: -0.078 (-0.106 to -0.050) 1998: -0.047 (-0.063 to -0.031) 2000: 0.033 (0.027 to 0.038) 2006: 0.032 (0.026 to 0.039) 2009: 0.000 (-0.003 to 0.004) 2017: 0.020 (0.016 to 0.023)  YLD attributable to AUD 1994: -6.760 (-8.554 to -4.966) 1998: -4.520 (-5.933 to -3.107) 2000: 2.194 (1.658 to 2.730) 2006: 4.226 (3.627 to 4.825) 2009: -0.387 (-0.559 to -0.216) 2017: 1.057 (0.957 to 1.158) | Study period: 1961-2019 |
| Zhang Z, Hu X, Zhang X, Zheng R. Do tougher drinking policies affect men's smoking behavior - Evidence from China. Soc Sci Med. 2024 348:116875. doi: 10.1016/j.socscimed.2024.116875. PMID: 38613870. | Longitudinal | Criminalization of drink-driving | 2011 | China | Current smokers (within last month), cigarettes smoked per day, drink frequency, alcohol intake (past week) | Policy led to a 2% decrease in the likelihood of smoking and a reduction of 0.92 in the number of cigarettes smoked per day. Policy led to reduction in probability of drinking more than three times a week by 3.27% and reduction in amount of alcohol intake per week by 18.06%. | Study period: 2010 and 2012 |

Note: parenthesis contain 95% confidence interval unless otherwise indicated.

## Appendix Figure 1: PRISMA flow diagram of alcohol control policy studies in China (original search October 2023)

Identification

References from other sources **(n = 21)**

Citation searching (n = 22)

WHO Country Studies Archive (n = 0)

References removed **(n = 2905)**

Duplicates identified manually (n = 38)

Duplicates identified by Covidence (n = 2867)

Marked as ineligible by automation tools (n = 0)

Included

Studies included in review **(n = 102)**

Of these, studies specific to China, Hong Kong and Taiwan **(n = 16 individual policy evaluations and 4 reviews)**

Studies screened **(n = 5041)**

Studies sought for retrieval **(n = 215)**

Studies assessed for eligibility **(n = 214)**

Studies excluded **(n = 4826)**

Studies not retrieved **(n = 1)**

Studies excluded **(n = 118)**

Wrong outcome (n = 25)

Wrong exposure (n = 38)

Wrong population (n = 2)

Wrong study design (n = 41)

Duplicate data from another study included (n = 4)

Wrong topic (not about alcohol control policies) (n = 5)

Data is aggregate for countries within and outside of WPR (n = 2)

Screening

References from databases/registers **(n = 7932)** (as **n = 7929** studies)

Embase (n = 3796)

MEDLINE (n = 3475)

EconLit (n = 661)

## Appendix 5: PRISMA checklist

See separate file

## References

1. Ezzati M, Lopez A, Rodgers A, Murray CJL. Comparative quantification of health risks. Global and regional burden of disease attributable to selected major risk factors. Geneva, Switzerland: World Health Organization; 2004.

2. Rehm J, Room R, Monteiro M, Gmel G, Graham K, Rehn T. Alcohol use. In: M. Ezzati ADL AR, & C.J.L. Murray, ed. Comparative quantification of health risks; Global and regional burden of disease attributable to selected major risk factors. Geneva: World Health Organization; 2004: 959-1108.

3. Rehm J, Kehoe T, Gmel G, Stinson F, Grant B, Gmel G. Statistical modeling of volume of alcohol exposure for epidemiological studies of population health: the US example. *Popul Health Metr* 2010; **8**: 3. doi: 10.1186/1478-7954-8-3

4. Shield K, Manthey J, Rylett M, et al. National, regional, and global burdens of disease from 2000 to 2016 attributable to alcohol use: a comparative risk assessment study. *Lancet Public Health* 2020; **5**(1): E51-E61. doi: Doi 10.1016/S2468-2667(19)30231-2

5. Murray CJL, Lopez AD. The global burden of disease: a comprehensive assessment of mortality and disability from diseases, injuries, and risk factors in 1990 and projected to 2020. Cambridge, MA: Published by the Harvard School of Public Health on behalf of the World Health Organization and the World Bank; Distributed by Harvard University Press; 1996.

6. World Health Organization. Global Health Estimates. 2023. <https://cdn.who.int/media/docs/default-source/gho-documents/global-health-estimates/gpe_discussion_paper_series_paper31_2001_age_standardization_rates.pdf> (accessed 08/12/2023).

7. United Nations. World Population Prospects: Data Booklet 2017 Revision. 2017. <https://www.un.org/development/desa/pd/sites/www.un.org.development.desa.pd/files/files/documents/2020/Jan/un_2017_world_population_prospects-2017_revision_databooklet.pdf> (accessed 08/12/2023).

8. Ahmad OB, Boschi-Pinto C, Lopez AD, Murray CJL, Lozano R, Inoue M. Age Standardization of Rates: A New WHO Standard2001. <https://cdn.who.int/media/docs/default-source/gho-documents/global-health-estimates/gpe_discussion_paper_series_paper31_2001_age_standardization_rates.pdf> (accessed 08/12/2023).

9. Gmel G, Shield KD, Frick H, Kehoe T, Gmel G, Rehm J. Estimating uncertainty of alcohol-attributable fractions for infectious and chronic diseases. *BMC Medical Research Methodology* 2011; **11**(1): 48.

10. Imtiaz S, Shield KD, Roerecke M, Samokhvalov AV, Lönnroth K, Rehm J. Alcohol consumption as a risk factor for tuberculosis: meta-analyses and burden of disease. *European Respiratory Journal* 2017; **50**(1): 1700216.

11. Rehm J, Probst C, Shield KD, Shuper PA. Does alcohol use have a causal effect on HIV incidence and disease progression? A review of the literature and a modeling strategy for quantifying the effect. *Popul Health Metr* 2017; **15**(1): 4. doi: 10.1186/s12963-017-0121-9

12. Samokhvalov A, Irving H, Rehm J. Alcohol consumption as a risk factor for pneumonia: a systematic review and meta-analysis. *Epidemiology and Infection* 2010; **138**(12): 1789-95.

13. Bagnardi V, Rota M, Botteri E, et al. Alcohol consumption and site-specific cancer risk: a comprehensive dose-response meta-analysis. *Br J Cancer* 2015; **112**(3): 580-93. doi: 10.1038/bjc.2014.579

14. Vieira A, Abar L, Chan D, et al. Foods and beverages and colorectal cancer risk: a systematic review and meta-analysis of cohort studies, an update of the evidence of the WCRF-AICR Continuous Update Project. *Annals of Oncology* 2017; **28**(8): 1788-802.

15. World Cancer Research Fund/American Institute for Cancer Research. Diet, nutrition, physical activity and cancer: a global perspective. Continuous Update Project expert report 2018. . London, United Kingdom, 2018.

16. Sun Q, Xie W, Wang Y, et al. Alcohol consumption by beverage type and risk of breast cancer: a dose-response meta-analysis of prospective cohort studies. *Alcohol and Alcoholism* 2020; **55**(3): 246-53.

17. World Health Organization. Global Information System on Alcohol and Health. Geneva, Switzerland: World Health Organization, 2023.

18. Samokhvalov AV, Irving H, Mohapatra S, Rehm J. Alcohol consumption, unprovoked seizures, and epilepsy: A systematic review and meta‐analysis. *Epilepsia* 2010; **51**(7): 1177-84.

19. Liu F, Liu Y, Sun X, et al. Race-and sex-specific association between alcohol consumption and hypertension in 22 cohort studies: A systematic review and meta-analysis. *Nutrition, Metabolism and Cardiovascular Diseases* 2020; **30**(8): 1249-59.

20. Rehm J, Shield KD, Roerecke M, Gmel G. Modelling the impact of alcohol consumption on cardiovascular disease mortality for comparative risk assessments: an overview *BMC Public Health* 2016; **16**: 363.

21. Roerecke M, Rehm J. The cardioprotective association of average alcohol consumption and ischaemic heart disease: a systematic review and meta-analysis. *Addiction* 2012; **107**(7): 1246-60.

22. Patra J, Taylor B, Irving H, et al. Alcohol consumption and the risk of morbidity and mortality from different stroke types - a systematic review and meta-analysis. *BMC Public Health* 2010; **10**(1): 258.

23. Larsson SC, Wallin A, Wolk A, Markus HS. Differing association of alcohol consumption with different stroke types: a systematic review and meta-analysis. *BMC medicine* 2016; **14**(1): 1-11.

24. Larsson SC, Drca N, Wolk A. Alcohol consumption and risk of atrial fibrillation: a prospective study and dose-response meta-analysis. *Journal of the American College of Cardiology* 2014; **64**(3): 281-9.

25. Roerecke M, Vafaei A, Hasan OSM, et al. Alcohol consumption and risk of liver cirrhosis: a systematic review and meta-analysis. *Am J Gastroenterol* 2019; **114**(10): 1574-86. doi: 10.14309/ajg.0000000000000340

26. Samokhvalov AV, Rehm J, Roerecke M. Alcohol consumption as a risk factor for acute and chronic pancreatitis: a systematic review and a series of meta-analyses. *EBioMedicine* 2015; **2**(12): 1996-2002.

27. World Health Organization. Global status report on alcohol and health 2018. 2018. <https://www.who.int/substance_abuse/publications/global_alcohol_report/en/> (accessed 11/14/2019).
